# Neuroprotection of *Gastrodia elata* polyphenols against H_2_O_2_-induced PC12 cell cytotoxicity by reducing oxidative stress

**DOI:** 10.3389/fphar.2022.1050775

**Published:** 2022-11-10

**Authors:** Weijian Tan, Qinhua Zheng, Kexin Feng, Xiaolin Feng, Wenting Zhong, Caiyu Liao, Shangjian Li, Yuntong Liu, Wenzhong Hu

**Affiliations:** ^1^ College of Pharmacy and Food Science, Zhuhai College of Science and Technology, Zhuhai, China; ^2^ College of Life Science, Jilin University, Changchun, China

**Keywords:** Gastrodia elata, polyphenols, alzheimer’s disease, oxidative stress, PC12 cell

## Abstract

It has been suggested that oxidative stress (OS) has a role in the development of aging and neurodegenerative disorders. Biological molecules are easily damaged by reactive oxygen species, which can ultimately result in necrotic or apoptotic cell death. Foods containing phytochemicals, such as phenolic compounds, may have potential preventive effects against several diseases, including alzheimer’s disease (AD), according to epidemiological and *in vitro* research. *Gastrodia elata* is a well-known homology of medicine and food plant that has been used for centuries in China and other East Asian countries to treat central nervous system disorders. In this study, we focused on the potential of the extract, *Gastrodia elata* polyphenols (GPP), for the prevention and treatment of AD. H_2_O_2_ induced PC12 cell damage was used to simulate the oxidative stress of AD. The effects of GPP on the injury model were evaluated by cell survival rate, lactate dehydrogenase (LDH), lipid peroxidation (MDA), production of intracellular antioxidant enzymes, reactive oxygen species (ROS), mitochondrial membrane potential (MMP), cellular inflammation level and apoptosis level. The results showed that GPP pretreatment had a protective effect by increasing cell viability, reducing lactate dehydrogenase infiltration, decreasing MDA and increasing intracellular antioxidant enzymes, diminishing reactive oxygen species production and decreasing mitochondrial membrane potential, reducing cell inflammation and decreasing apoptosis. Accordingly, it is suggested that GPP possessed promising neuroprotective benefits which enabled the prevention or therapeutic implementation of AD along with serving as a reference towards the exploitation of functional foods or drugs derived from *Gastrodia elata*.

## 1 Introduction

Aging and neurodegenerative disorders are greatly influenced by oxidative stress (Trushina and McMurray, 2007). The growth of neurodegenerative illnesses and the aging process are both correlated with the gradual accumulation of ROS, which causes the breakdown of macromolecules like proteins, DNA, and lipids. Numerous negative consequences, including decreased ATP synthesis and mitochondrial malfunction, can be brought on by excess ROS. The regular operation of neuronal cells and their response to stress during aging and AD development are impacted by high or low mitochondrial ATP generation. Mitochondrial enlargement and consequent cell death are caused by mitochondrial permeability transition pores (Bhat et al., 2015). DNA oxidative damage is another side effect of oxidative stress that can result in cellular malfunction and death. Alzheimer’s disease (AD) is one of the most common progressive age-related neurodegenerative disorders in which patients suffer from persistent memory and cognitive decline, severely affecting their quality of life (Zhao, 2020). The number of people suffering from AD is currently over 50 million worldwide and is expected to exceed 150 million by 2050 as the aging process accelerates in all countries (Panza et al., 2019). Countries all over the world are increasing funds to support the development of AD drugs, but currently available anti AD drugs can only improve patients’ memory and cognitive ability, delay the onset of disease, but cannot cure AD, and clinical treatment of AD still has great challenges (Martin et al., 2013). In recent years, clinical drug trials targeting the inhibition of Aβ proteins or abnormal Tau proteins have failed to achieve the expected efficacy, casting a cloud over the development of anti-AD drugs (Castello et al., 2014; Mullard, 2021). The oxidative stress theory links mitochondrial dysfunction and increased ROS to the development of AD, provides an explanation for the outcome of the multifactorial development. Mitochondrial dysfunction has been demonstrated in AD, with mitochondrial splitting, fusion, and other abnormalities have been observed in patients before the onset of amyloid plaque and neuronal tangle injury (Trushina and McMurray, 2007; Mosconi et al., 2008). Mitochondrial dysfunction promotes the accidental generation of ROS, and oxidative stress is also associated with an imbalance between the production and elimination of ROS, the excess of which oxidizes all major biological macromolecules, including nucleic acids (DNA and RNA), proteins, and lipids. At the same time, studies have shown that ROS can promote the formation of Aβ aggregates and abnormal phosphorylation of Tau protein, further aggravating the pathogenesis of AD (Nunomura et al., 2001; Butterfield et al., 2007; Wang et al., 2014).

*Gastrodia elata* (*G.elata*) is a well-know herbal medicine, which has been used in China and other eastern countries for centuries to treat central nervous system diseases, such as headache, dizziness, convulsion, epilepsy and stroke. As a parasitic plant, the whole plant of *Gastrodia elata* has no chloroplast and the medicinal part is its rhizome. Since 1950, more than 80 compounds have been isolated from *G. elata* rhizomes, including phenolic, polysaccharides, organic acids, sterols, etc. The phenolic components of Gastrodin are considered to be its main active constituents, of which Gastrodin as a phenolic glycoside whose content has been established by the Chinese Pharmacopoeia as the most important phytochemical marker for quality standards of *G. elata* rhizomes (Huang et al., 2015; Liu et al., 2018; Ye et al., 2019). Studies have been widely reported on the neuroprotective effects of Gastrodin such as anticonvulsant, antiheadache, anxiolytic and antidepressant (Lin et al., 2016; Yan et al., 2019; Yang et al., 2021). However, the pharmacological effects of *G. elata* polyphenols (GPP) have rarely been reported. Polyphenols are natural antioxidants that widely found in plants. It exhibits potential for the prevention and treatment of AD because of its excellent antioxidant, anti-inflammatory, neuroprotective, and gut health promoting properties (Rajasekhar and Thimmaiah, 2018). The neuroprotective effects of *G. elata*, a traditional herb, have been demonstrated; therefore, the polyphenolic components of *G. elata* may have significant anti-AD potential. The PC12 cell line, which was first generated from rat pheochromocytoma, has been used frequently as a neuronal model for research on AD and other neurodegenerative diseases since it largely resembles main neurons (Xu et al., 2016; Zhang et al., 2020; Li et al., 2022). H_2_O_2_ exposure causes apoptosis and other alterations in cultured neuronal cells that mirror those in the brains of AD patients (Wojtunik-Kulesza et al., 2016). In this study, we used PC12 cells with H_2_O_2_-induced injury to construct a cell model of AD oxidative stress injury to investigate the protective potential of GPP against the injury model. We hope to provide a reference for the development of GPP drugs for the prevention and treatment of AD.

## 2 Material and methods

### 2.1 Materials

*G. elata* (20220415) was purchased from Baoyuantang Pharmaceutical Co., Ltd. (Guangdong, China). Gallic acid reference substance (B20851) was purchased from Shanghai Yuanye Biotechnology Co., Ltd. (Shanghai, China). Folin Ciocalteu reagent, FeCl_3_, Lead acetate and H_2_O_2_ were purchased from Sinopharm Chemical Agent Co., Ltd. (Shanghai, China). RMPI 1640 was purchased from Gibco Co., Ltd (California, United States). Fetal Bovine Serum (FBS) was purchased from Procell Life Science&Technology Co., Ltd. (Wuhan, China). LDH (A020-2), MDA (A003-4), CAT (A007-1), GSH-Px (A006-2), NO (A013-2) were purchased from Nanjing Jiancheng Co., Ltd. (Beijing, China). Cell Counting Kit-8 (CCK8, C0038) was purchased from Beyotime Biotechnology Co., Ltd. (Shanghai, China). JC-1 (M8650) kit, DAPI solution (10 μg/ml, C0065), 4% tissue cell fixative (P1110), DCFH-DA probe kit (CA1410), Annexin V/PI kit (CA1020), Caspase-3 (BC3830) and Caspase-9 (BC3890) kits were purchased from Solarbio Co., Ltd. (Beijing, China). Rat TNF-α ELISA KIT (RX302058R) and Rat IL-6 ELISA KIT (RX302856R) were purchased from Ruixin Biotechnology Co., Ltd (Quanzhou, China).

### 2.2 Extraction, isolation, and purification of *Gastrodia elata* polyphenols

**FIGURE 1:**
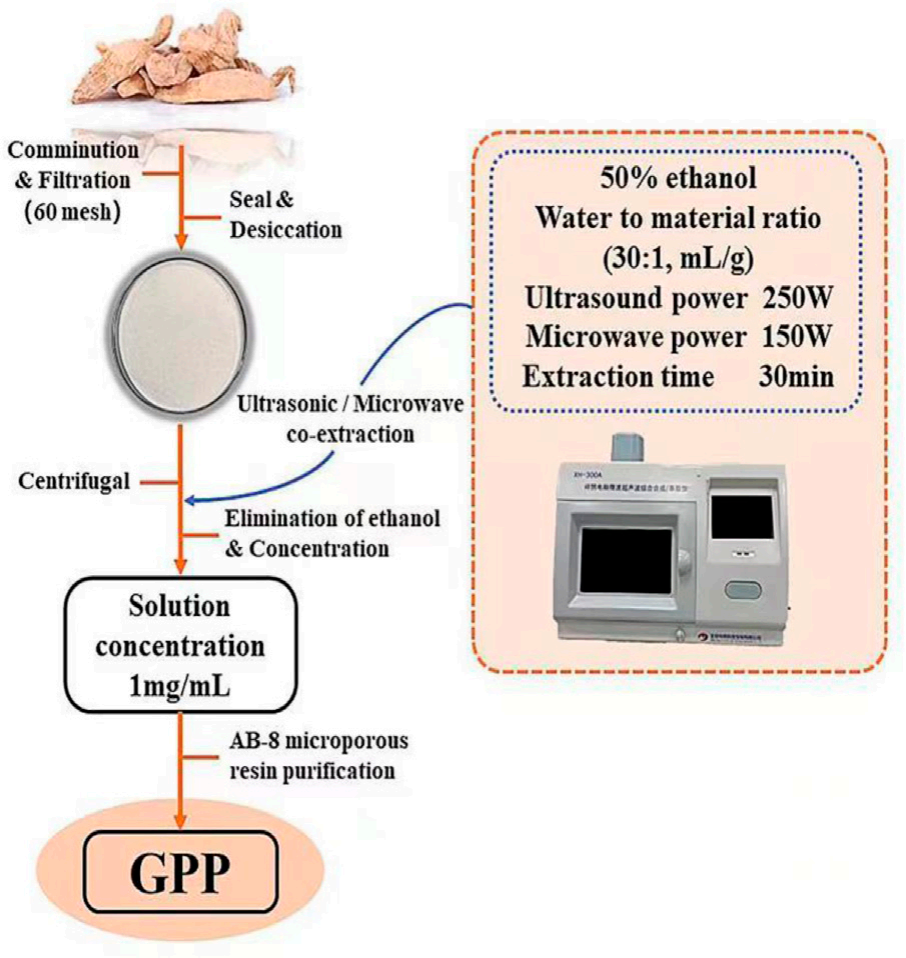
The extraction, isolation, and purification procedure of GPP.

*G. elata* was obtained from Zhaotong City, Yunnan Province, China, which authenticated by Zhang Runrong, senior engineer of traditional Chinese medicine, College of Pharmacy and Food Science, Zhuhai College of Science and Technology, Zhuhai, Guangdong Province, China. The dried *G. elata* rhizomes were crushed and sieved, and GPP was extracted using an ultrasonic/microwave co-extractor. Extraction parameters were ultrasonic power 250 W, microwave power 150 W, extraction time 30 min, material-liquid ratio 1:30 (Solvent: 50% ethanpl). Cooling at room temperature, centrifugation for 10 min (5000 rpm/min), transfer of supernatant, and purification of the extract by AB-8 macroporous sorbent resin. The purified extract was concentrated under reduced pressure using a vacuum rotary evaporator, then freeze-dried to obtain GPP and stored at −20°C until use (Figure 1). GPP was determined by measuring the concentration of polyphenols (represented by gallic acid equivalents, i.e., GAE) in aqueous solution by Folin-Ciocalteu reagent (using gallic acid as a control), which is a mixture of phosphotungstic acid and phosphomolybdic acid was prepared. The addition of Folin-Ciocalteu reagent to the polyphenol solution resulted in the formation of chromophore compounds, which have maximum absorbance at 760 nm (Singleton et al., 1999). After measuring the polyphenol content, the polyphenol yield was analyzed and the yield of purified GPP was calculated to be 63.97% ± 0.67%. The equation for the yield of polyphenols is as follows: **Y** is the yield of polyphenols (%), **V** is the total volume of the sample solution (ml), **C** is the concentration of polyphenols (mg GAE/mL), **D** is the dilution factor, and **m** is the mass of the sample weighed (g).

$$Y\;\left(\%\right)=\frac{V\times C\times D}{m\times 10^{3}}\times 100$$

### 2.3 Determination of UV spectra

The above GPP samples and the Gallic acid Reference substance were prepared as 0.15 mg/ml solution and scanned at full wavelength from 0 to 400 nm using distilled water as control.

### 2.4 Determination of infrared spectra

Completely dried KBr was mixed with the above GPP samples and ground in an agate mortar at a mass ratio of 100:1 to 200:1 in a sufficiently dry environment. The sections were pressed and scanned with an infrared spectrometer in the range of 500 cm^−1^ to 4000 cm^−1^.

### 2.5 Determination of physicochemical properties of *Gastrodia elata* polyphenols

The polyphenolic compounds in the purified products were identified by the color development reaction of GPP with FeCl_3_ and lead acetate, respectively, according to a standard procedure reported in the literature (Mondal et al., 2017). Briefly, 10 mg of GPP was dissolved in 100 ml of 70% ethanol solution and several drops of 1% FeCl_3_ solution were added to the solution to observe the color change of GPP solution; 10 mg of GPP was dissolved in 100 ml of 70% ethanol solution and neutral lead acetate solution was added to the solution to observe the color change of GPP solution.

#### 2.6 Cell culture

In this work, PC12 cells were obtained at the Cell Resource Center of Shanghai Institute of Biological Science, Chinese Academy of Science (Shanghai, China). Briefly, PC12 cells were cultured at 37°C in a humidified atmosphere of 5% CO_2_ and 95% air in RMPI 1640 medium supplemented with 10% fetal bovine serum (FBS) and 0.1% penicillin/streptomycin. Cells were fed every 3 days and sub-cultured after reaching 80%–90% fusion in 75 cm^2^ cell culture flasks.

This work later divided PC12 cells into the following five groups: control group, H_2_O_2_ group, GPP low dose group (50 μg/ml), GPP medium dose group (100 μg/ml) and GPP high dose group (200 μg/ml). After pretreatment with or without a certain concentration of GPP for 24 h, the cells were exposed to H_2_O_2_ (250 μM) for 24 h. Cells or media were harvested for the assay.

### 2.7 Cell Counting Kit-8 assay

The CCK-8 cell viability assay is a sensitive measurement based on the WST-8 reagent. WST-8 is reduced by dehydrogenase in cells to a highly water-soluble yellow formazan dye, and the amount of formazan produced is proportional to the number of living cells to reflect the cell proliferation status. PC12 cells (1×10^5^ cells/mL in 96-well plates) were cultured at 37°C. After pretreatment with or without the addition of GPP for 24 h, cells were exposed to H_2_O_2_ (250 μM) for 24 h. The cell culture medium was then discarded. 100 μl of newly configured medium containing 10 μl of CCK-8 solution was added to each well, incubated for 1 h at 37°C, and the absorbance at 450 nm was measured using a microplate reader Epoch (Bio-Tek, Georgia, United States). Cell proliferation results were expressed as the change in absorbance of the sample relative to the absorbance of the control cells.

### 2.8 Determination of lactate dehydrogenase leakage, MDA, GSH-Px, SOD and CAT content

PC12 cells (1×10^5^ cells/mL in 6-well plates) were cultured at 37°C, pretreated by adding different doses of GPP for 24 h, and then exposed to H_2_O_2_ (250 μM) to treat the cells for 24 h. Cell cultures were collected, and cells were collected using a cell scraper. Then, cells were sonicated in a 200W 4°C ice water bath for 5 min and centrifuged at 1000 rpm and 4°C for 5 min to collect the supernatant of the cell. The amount of LDH leaked from the cell culture medium was determined using a commercially available LDH assay kit, and the levels of MDA, GSH-Px, SOD, and CAT in the cells supernatant were measured according to the instructions of the kit.

#### 2.9 ROS assay

The 2′,7′-dichlorofluorescein diacetate (DCFH-DA) probe with cell membrane permeability is enzymatically cleaved to DCF upon entry into cells, where it accumulates and fluoresces, thus reflecting ROS levels. For this work, PC12 cells (1×10^5^ cells/ml) were incubated into 6-well plates and treated according to the grouping described above. At the end of the treatment, the medium was discarded and 2 ml of DCFH-DA probe diluted at a ratio of 1:1000 was added to each well, and the serum-free medium was washed three times. The fluorescence intensity of 480 nm excitation and 525 nm emission was examined using a flow cytometer Cyto FLEX (Beckman, Indianapolis, United States). Meanwhile, the fluorescence intensity was observed using a fluorescent inverted microscope IX73 (Olympus, Tokyo, Japan).

### 2.10 IL-6, TNF-α and NO assay

The secretion of IL-6, TNF-α and NO reflects the level of inflammation in the injury model. Among them, IL-6 and TNF-α levels were measured by enzyme-linked immunosorbent assay (ELISA). PC12 cells (1×10^5^ cells/ml) were incubated into 6-well plates and treated as grouped as described above. The cell culture supernatant was then collected at the end of the treatment and the levels of IL-6, TNF-α and NO were determined according to the instructions of the kit.

### 2.11 JC-1 and DAPI assay

In this work, the mitochondrial membrane potential (MMP) was analyzed using the JC-1 probe. PC12 cells (1×10^5^ cells/mL) were incubated into 6-well plates and treated as grouped above. At the end of treatment, the medium was discarded, and the JC-1 probe was added after washing 3 times with PBS and stained at 37°C for 20 Min. Fluorescence intensity was observed using a fluorescent inverted microscope. 4′,6-diamidino-2-phenylindole (DAPI) is a fluorescent dye that binds vigorously to DNA, and the fluorescence intensity of cells observed by DAPI staining. PC12 cells (1×10^5^ cells/ml) were incubated in 6-well plates, and after the same grouping and treatment as above, the cells were fixed with 4% paraformaldehyde for 20 min, washed 3 times with PBS and incubated with 10 μg/ml DAPI solution for 20 min. The fluorescence intensity was observed using a fluorescence inverted microscope.

### 2.12 Apoptosis assay

Apoptosis was determined using the FITC (fluorescein isothiocyanate) Membrane Linker V (Annexin V) Apoptosis Detection Kit. PC12 cells (1×10^5^ cells/ml) were incubated into 6-well plates and treated as grouped above. At the end of the treatment, cells under different treatments were separated using trypsin without EDTA and washed with PBS. After centrifugation, plasma cells were suspended in binding buffer, Annexin V-FITC staining solution (5 μl) and PI staining solution (5 μl) were added. After incubation for 10 min at room temperature, cells were analyzed by flow cytometry.

### 2.13 Caspase 3, Caspase 9 assay

The activation of the Caspase protein family reflects the level of apoptosis. PC12 cells (1×10^5^ cells/ml in 6-well plates) were cultured at 37°C and treated as grouped above. At the end of treatment, cells under different treatments were separated using trypsin without EDTA and washed with PBS. After centrifugation, 100 μl of cell lysate was added for 15 min at 4°C. Cells were centrifuged at 15,000 rpm for 15 min. Cell supernatants were gathered. The levels of Caspase 3 and Caspase 9 proteins in the supernatant were determined according to the instructions of the commercially available kits.

### 2.14 Statistical analysis

Data are shown as mean ± SEM, and statistical analysis was performed using Origin 2021, Graphpad Prism 8, and EXCEL 2019. To compare the differences between multiple groups, a one-way analysis of variance (ANOVA) was performed in this software. *p* < 0.05 indicates statistical significance.

## 3 Results

### 3.1 The analysis of UV spectra

**FIGURE 2:**
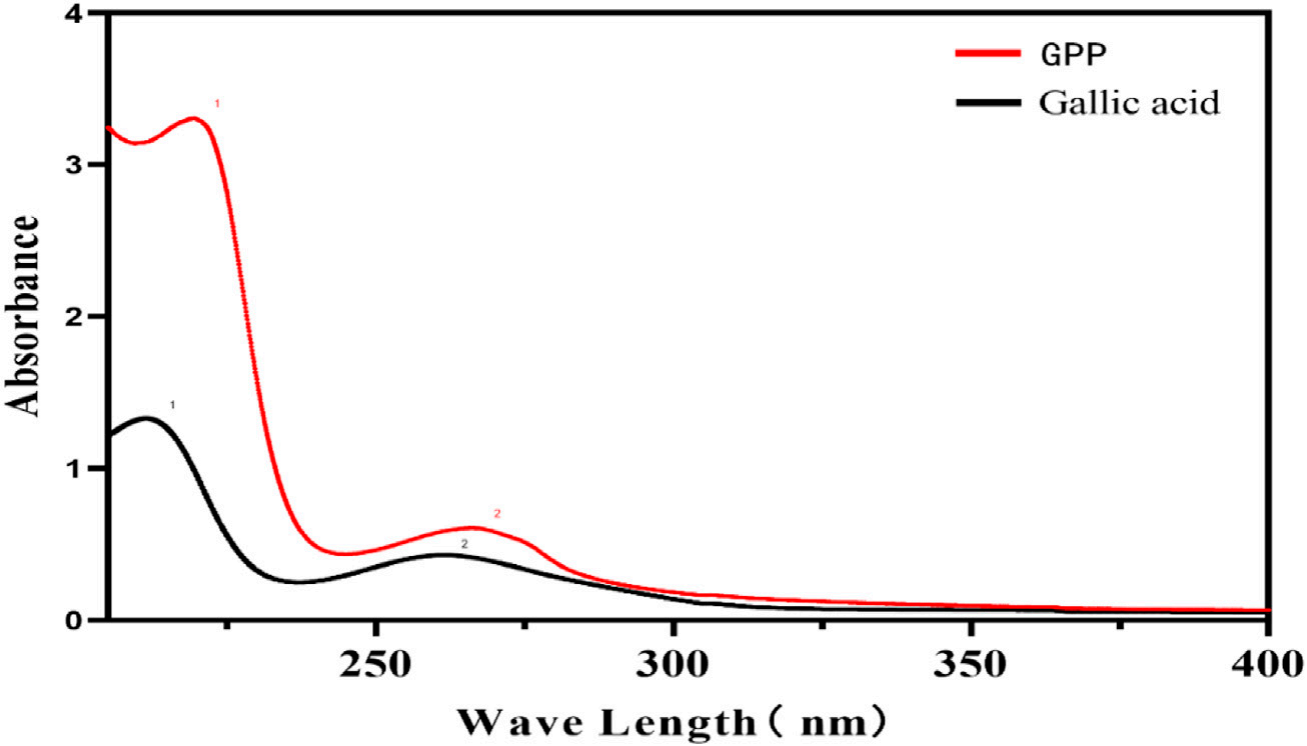
UV spectra of GPP and Gallic acid reference substance.

The UV spectra of GPP and Gallic acid reference substance were scanned as shown in Figure 2, and the results showed that GPP has a characteristic absorption peak similar to that of Gallic acid, with the maximum absorption wavelength around 210 nm and 270 nm. The absorption peak at 270 nm is a typical absorption peak of polyphenols, so it is assumed that GPP may contain Gallic acid or a functional group similar to Gallic acid.

### 3.2 The analysis of IR

**FIGURE 3:**
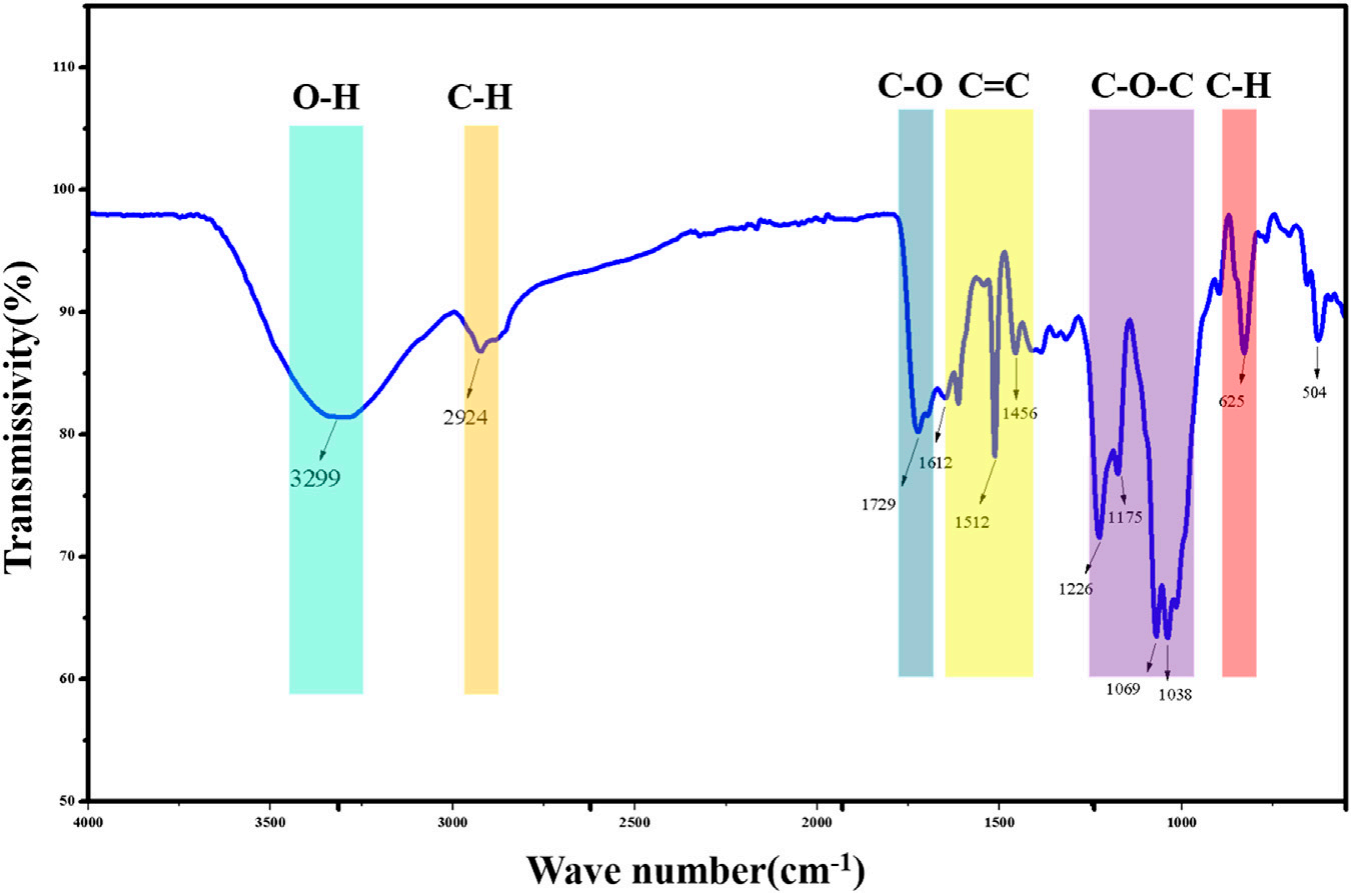
FT-IR results of GPP.

The FT-IR spectrum of GPP is shown in Figure 3. The intense peak is located at 3299 cm^−1^ and considered as O-H stretching vibration of polyphenol benzene ring. 2924 cm^−1^ is the asymmetric stretching vibration of polyphenol C-H bond (Ismet et al., 2010). 1729 cm^−1^ is considered as the stretching vibration of carbonyl C=O. Weaker stretching vibration is observed in the range of 1612 cm^−1^-1456 cm^−1^ and considered as C=C stretching vibration of polyphenol aromatic ring skeleton (Sm et al., 2021). The absorption peaks observed in the range 1226 cm^−1^-1038 cm^−1^ were considered as asymmetric stretching vibrations of C-O-C. 625 cm^−1^ was considered as external bending vibrations of aromatic C-H (Dilek et al., 2011). In conclusion, the IR spectral features of GPP are consistent with those of polyphenolic compounds in general, and GPP does contain polyphenolic compounds.

### 3.3 Physicochemical property analysis of *gastrodia elata* polyphenols

The GPP solution after the addition of FeCl_3_ turned into a dark brown solution after shaking; adding neutral lead acetate solution to the GPP solution immediately produced a precipitate, which gradually increased with the increase of neutral lead acetate solution (Table 1). The above two identification reactions are consistent with the reaction characteristics of polyphenolic compounds, indicating that GPP does contain polyphenolic compounds.

**TABLE 1:** Polyphenol identification reaction of GPP.

Testing indicators	GPP
Ferric chloride experiment	**+**
Lead acetate experiment	**+**

Note:+. Positive.

### 3.4 H_2_O_2_ model establishment and GPP mitigated the H_2_O_2_-Treated PC12 cell viability loss

**FIGURE 4:**
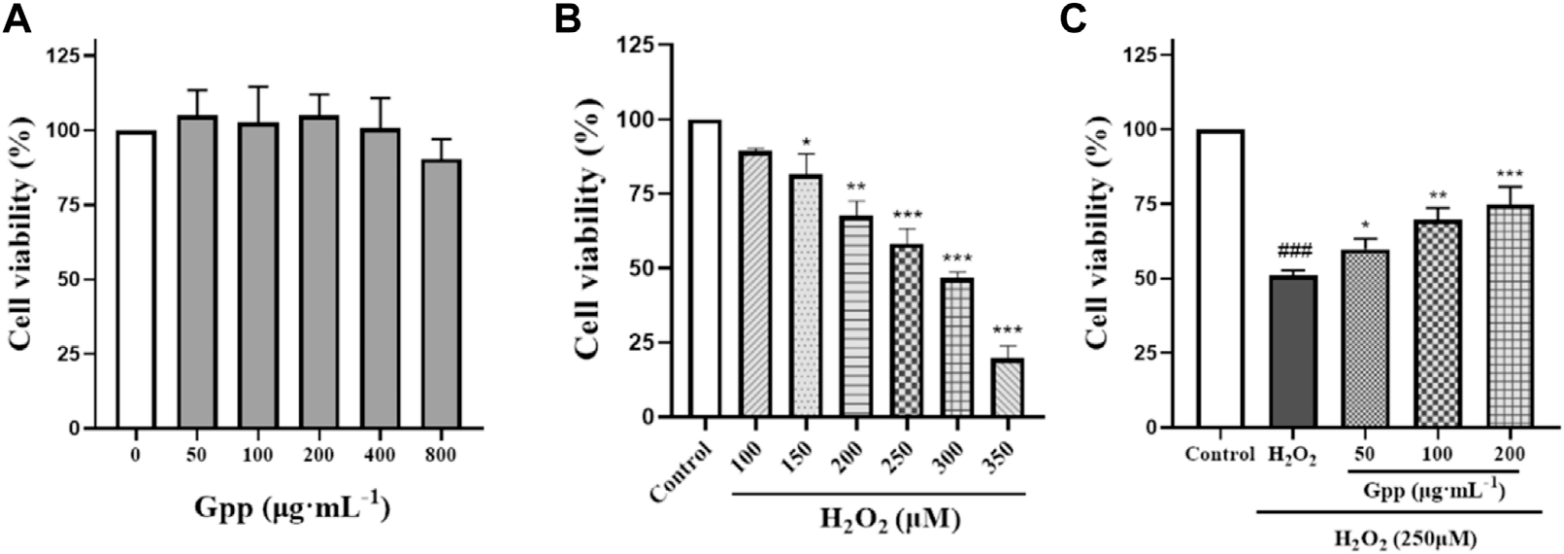
Effect of different concentrations of GPP pretreatment on PC12 cell survival **(A)** Effect of different concentrations of H_2_O_2_ treatment on PC12 cell survival **(B)** Protective effect of different concentrations of GPP pretreatment on H_2_O_2_ 250 μM induced PC12 damage **(C)**. *p < 0.05, **p < 0.01, ***p < 0.001, versus H_2_O_2_ group, ^#^ *p* < 0.05, ^##^ *p* < 0.01, ^###^ *p* < 0.001versus Control.

To establish a model of oxidative stress injury in Alzheimer’s disease, H_2_O_2_ was used to induce PC12 cell death in this study. The optimal H_2_O_2_ concentration that induced a 40%–60% reduction in PC12 cell viability was determined by the CCK-8 method. Firstly, the effect of different concentrations of GPP treatment on PC12 cells was investigated, as shown in Figure 4A. Compared with the control group, pretreatment with 50–800 μg/ml GPP for 24 h did not significantly affect the cell viability of PC12 cells (*p* > 0.05), and the viability of PC12 cells gradually decreased in a concentration-dependent manner with increasing H_2_O_2_ concentration (Figure 4B), and the viability of PC12 cells induced by 250 μM H_2_O_2_-induced cell survival in the experimental group was close to IC50, which helped to simulate oxidative stress-mediated PC12 cell injury.

In addition, GPP reversed the H_2_O_2_-mediated decrease in PC12 cell viability (Figure 4C), which was significantly reduced after 250 μM H_2_O_2_ treatment compared to the control (51.04 ± 1.73%, *p* < 0.001), while PC12 cell survival was significantly higher and dose-dependent after 24 h pretreatment with GPP (respectively 59.83 ± 3.46%, 69.80 ± 3.88%, 74.69 ± 6.11%, *p* < 0.05), indicating that GPP has a potent protective effect on PC12.

### 3.5 *Gastrodia elata* polyphenols alleviates H_2_O_2_-Induced lipid peroxidation and increases the level of endogenous antioxidant enzymes in cells

**FIGURE 5:**
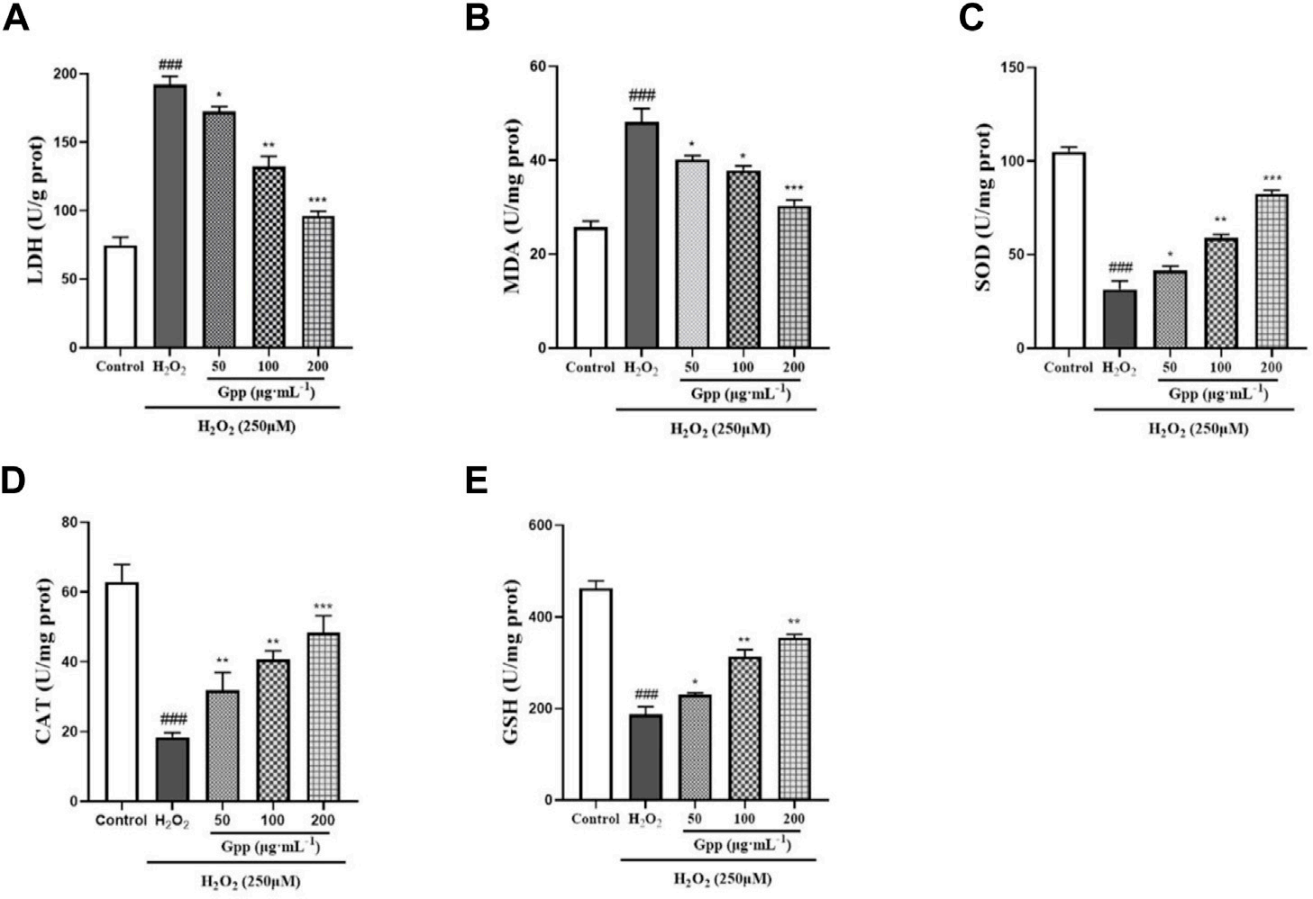
Effect of GPP pretreatment on H_2_O_2_-induced damage to LDH **(A)**, MDA **(B)**, SOD **(C)**, CAT **(D)**, and GSH-Px **(E)**. *p < 0.05, **p < 0.01, ***p < 0.001, versus H_2_O_2_ group, ^#^ *p* < 0.05, ^##^ *p* < 0.01, ^###^ *p* < 0.001versus Control.

As shown in Figures 5A,B, LDH leakage was significantly higher in the H_2_O_2_ group (192 ± 6.18 U/g prot; *p* < 0.001) compared with the control group (74.71 ± 5.93 U/g prot). Nevertheless, 50, 100 and 200 μg/ml pretreatment significantly reduced LDH leakage levels in the injury model compared to the H_2_O_2_ group (172.4 ± 3.64 U/g prot, 132.5 ± 7.23 U/g prot and 96.13 ± 3.50 U/g prot, respectively; *p* < 0.05). Compared to the control group (25.72 ± 1.28 U/mg prot), cellular MDA levels were significantly higher in the H_2_O_2_ group (48.14 ± 2.86 U/g prot; *p* < 0.001), indicating that injury caused lipid peroxidation in PC12 cells. However, 50, 100 and 200 μg/ml pretreatment significantly reduced the injury compared to the H_2_O_2_ group MDA levels (40.13 ± 0.82 U/mg prot, 37.83 ± 0.94 U/mg prot and 30.28 ± 1.23 U/mg prot, respectively; *p* < 0.05). Meanwhile, H_2_O_2_ treatment all reduced cellular endogenous antioxidant enzymes SOD, CAT, and GSH-Px levels to varying degrees, suggesting that the injury model caused oxidative stress. Compared with the model group, 50, 100 and 200 μg/ml GPP pretreatment significantly increased SOD, CAT, and GSH-Px levels in the injury model (Figures 5C–E). In conclusion, GPP pretreatment dose-dependently increased the levels of endogenous antioxidant enzymes in PC12 cells and attenuated the oxidative stress damage caused by H_2_O_2_.

### 3.6 *Gastrodia elata* polyphenols attenuates H_2_O_2_-Induced production of elevated ROS and levels of neuroinflammation

**FIGURE 6:**
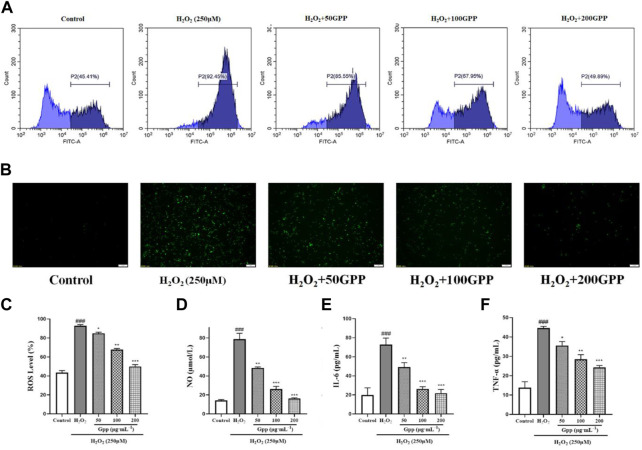
Effect of GPP pretreatment on ROS and neuroinflammation in injury models. Where **(A)** is the fluorescence intensity of ROS probe detected using flow cytometry, **(B)** is the fluorescence intensity of cellular ROS detected using fluorescence inverted microscopy, and **(C)** Statistical results of fluorescence intensity based on flow cytometry detection; Effect of GPP pretreatment on NO **(D)**, IL-6 **(E)**, and TNF-α **(F)** secretion in injury models. *p < 0.05, **p < 0.01, ***p < 0.001, versus H_2_O_2_ group, ^#^ *p* < 0.05, ^##^ *p* < 0.01, ^###^ *p* < 0.001 versus Control.

To quantitatively demonstrate the effect of GPP on H_2_O_2_-mediated elevation of ROS levels, flow cytometry assay with fluorescence inverted microscopy was performed by DCFH-DA probe staining. As shown in Figures 6A–C, 250 μM H_2_O_2_ treatment significantly increased the fluorescence intensity of the cells (92.19 ± 0.23%, *p* < 0.001) compared to the control (44.87 ± 0.87%). This suggests that H_2_O_2_ treatment caused an increase in cellular ROS levels, and 50, 100 and 200 μg/ml pretreatment significantly reduced the damage model ROS levels compared to the model group (85.5 ± 0.58%, 68.09 ± 0.83%, 50.59 ± 1.19%, respectively, *p* < 0.05). The results observed using fluorescence inverted microscopy were also consistent with the flow cytometry assay results.

To investigate whether the protective effect of GPP was mediated by alleviating the level of cellular inflammation, the levels of NO, IL-6 and TNF-α secretion in PC12 cells treated with H_2_O_2_ or GPP were analyzed in this study. As shown in Figures 6D–F, compared with the control group, 250 μM H_2_O_2_ treatment significantly increased the secretion of NO,IL-6 and TNF-α in PC12 cells, while GPP dose-dependently attenuated the secretion levels of H_2_O_2_-induced hyperinflammatory factors compared with the model group, effectively improving the inflammatory status of the cells (*p* < 0.05).

### 3.7 *Gastrodia elata* polyphenols shows a protective effect against H_2_O_2_-induced apoptosis and Ameliorates the reduction of mitochondrial membrane potential

**FIGURE 7:**
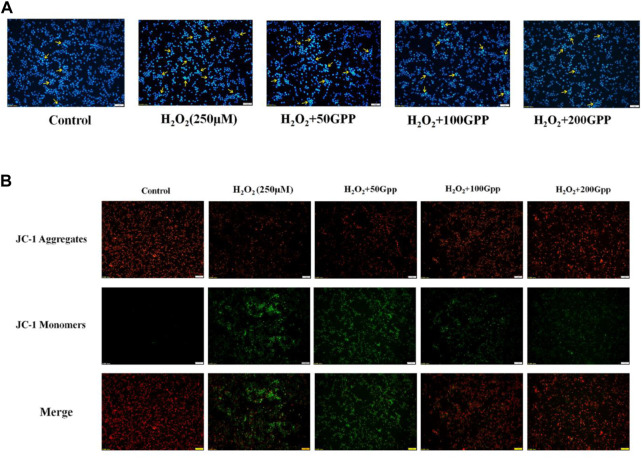
Effect of GPP pretreatment on apoptosis **(A)** and mitochondrial membrane potential **(B)** in injury models.

DAPI is a blue fluorescent dye that can penetrate cell membranes. When combined with double-stranded DNA, it can produce more than 20 times stronger fluorescence than DAPI itself. It is used for apoptosis detection because apoptotic vesicles that undergo nuclear condensation will emit a stronger blue fluorescence. As shown in Figure 7A, after H_2_O_2_ induction, PC12 cells showed a significant fluorescence enhancement and strong blue-white fluorescent cells were observed (shown by yellow arrows). Compared with the model group, the 50, 100, and 200 μg/ml GPP pretreatment groups showed a dose-dependent attenuation of fluorescence intensity, indicating that GPP pretreatment attenuated the H_2_O_2_-induced apoptosis.

A decrease in MMP is considered to be a marker of early apoptosis. In this study, we used JC-1 fluorescent probe to detect cellular MMP. In normal mitochondria, JC-1 aggregates in the mitochondrial matrix to form a polymer and emits red fluorescence; when the mitochondrial membrane is broken, JC-1 can only exist in the cytoplasm as a monomer and produces green fluorescence. As shown in Figure 7B, the PC12 cells in the control group survived well, and thus the fluorescence emitted was mainly red. Compared with the control group, the model group produced a large amount of green fluorescence, indicating that H_2_O_2_ treatment caused a decrease in cellular MMP and the integrity of mitochondria was disrupted. Compared with the model group, the 50, 100, and 200 μg/ml GPP pretreatment groups showed a dose-dependent attenuation of the H_2_O_2_-induced MMP reduction, with the 100 and 200 μg/ml GPP treatment groups restoring the cells to a level dominated by red fluorescence.

### 3.8 Quantification of H_2_O_2_-Induced apoptosis attenuated by *Gastrodia elata* polyphenols

**FIGURE 8:**
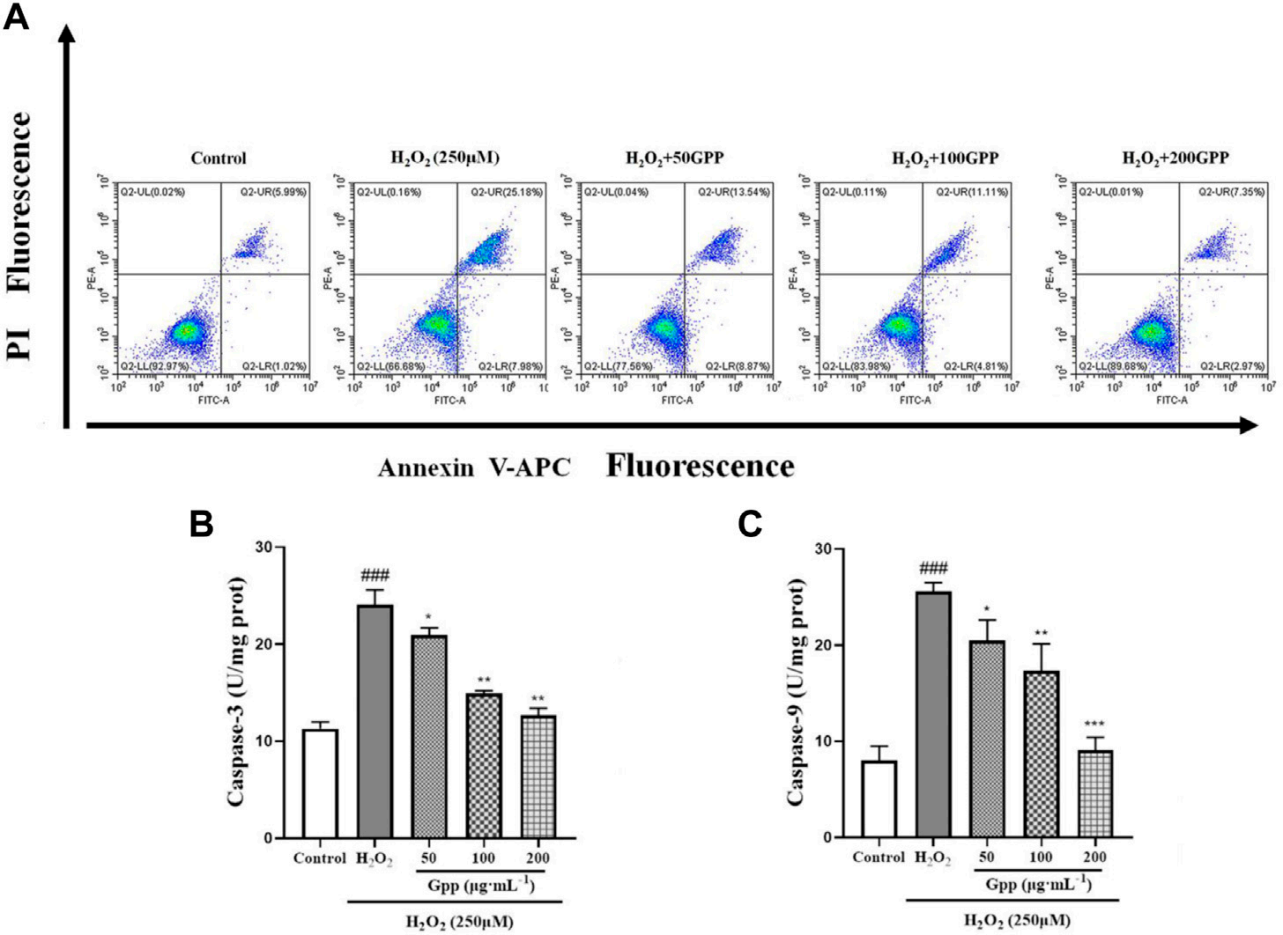
Quantitative analysis of GPP pretreatment on apoptosis in injury models, where **(A)** is the rate of apoptosis detected using flow cytometry, **(B)** is the result of Caspase-3 protein content measurement, and **(C)** is the result of Caspase-9 protein content measurement. *p < 0.05, **p < 0.01, ***p < 0.001, versus H_2_O_2_ group, ^#^ *p* < 0.05, ^##^ *p* < 0.01, ^###^ *p* < 0.001versus Control.

To quantitatively demonstrate the effect of GPP on H_2_O_2_-mediated apoptosis, the rate of apoptotic cells was detected by Annexin V/PI staining and flow cytometry. According to Figure 8A, the apoptosis rate in the control group was 6.48 ± 0.58% of the total cells. However, the apoptosis rate in the H_2_O_2_-treated model group was significantly increased to 35.64 ± 2.55%; GPP (50,100,200 μg/ml) pretreatment significantly reduced H_2_O_2_-induced apoptosis by 24.92 ± 2.25%, 16.20 ± 1.38%, and 11.68 ± 1.18%, respectively (*p* < 0.05).

Activation of the Cysteine aspartic acid specific protease (Caspases) is one of the hallmarks of apoptosis. In the present study, the expression of Caspase 3 and Caspase 9 proteins was examined in PC12 cells, which are involved in the recruitment and execution of apoptosis, respectively. As shown in Figures 8B,C. In the results of Caspase 9 protein assay, the protein expression was significantly higher in the model group (25.60 ± 0.90 U/mg prot, *p* < 0.001) compared to the control group (8.01 ± 1.49 U/mg prot). However, pretreatment with GPP (50,100,200 μg/ml) significantly ameliorated H_2_O_2_-induced elevated Caspase 9 protein expression by 20.50 ± 2.11 U/mg prot, 17.33 ± 2.78 U/mg prot, and 9.10 ± 1.31 U/mg prot, respectively, compared to the model group (*p* < 0.05). The same was found in the results of Caspase 3 assay (*p* < 0.05). The above results demonstrate that GPP pretreatment exerts a protective effect by alleviating apoptosis.

## 4 Discussion

**FIGURE 9:**
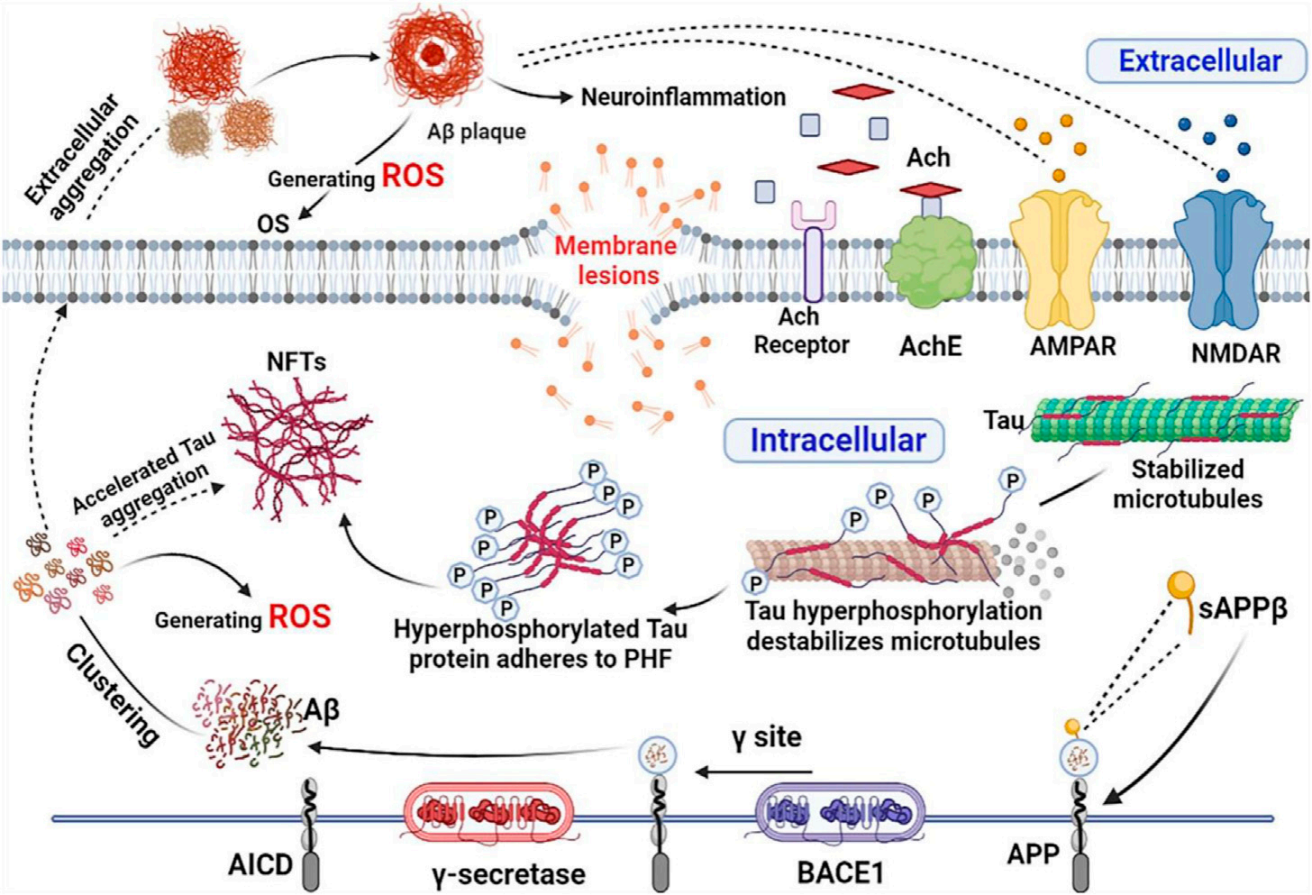
Oxidative stress combined with Aβ, abnormal Tau and neuroinflammation contribute to the development of AD. The figure was created employing online software named BioRender.

Oxidative stress refers to the steady-state degree of oxidative damage in cells, tissues, or organs brought on by an imbalance between the mechanisms that produce ROS, cellular oxidants, and antioxidants. With aging, oxidative damage and susceptibility to oxidative stress increase. The brain is the organ with the highest oxygen consumption in the body, which can account for more than 30% of the body’s oxygen consumption at the same moment, and this process inevitably generates a large amount of ROS; compared to other organs, the level of endogenous antioxidant enzyme production in the brain is relatively insufficient, so oxidative stress causes massive damage to nerve cells and induces AD. The pathways and mechanisms of oxidative stress damage include lipid peroxidation, protein oxidation, DNA peroxidation and NA oxidation (Srebro et al., 2008). Oxidative stress accelerates the aggregation of Aβ and induces the extracellular aggregation of Aβ to form Aβ plaques (Butterfield and Lauderback, 2002); accelerates the abnormal aggregation of Tau proteins (Su et al., 2010), which induces a complex cascade of neuroinflammatory responses, microglia activation, cytokine release and astrocyte proliferation, and these processes in turn promote the generation of ROS. Therefore, it is reasonable to believe that oxidative stress penetrates into the pathogenesis of AD and contributes to its formation through the malignant promotion of mechanisms such as combined Aβ aggregation and aberrant Tau proteins (Figure 9). H_2_O_2_ has been utilized frequently as an inducer of oxidative stress in *in vitro* models since it is the principal component of ROS that can cause apoptosis (Hwang and Yen, 2011). The CCK8 assay used in the current investigation demonstrated that pretreatment with 50, 100, and 200 g/ml GPP had a concentration-dependent protective effect against H_2_O_2_-induced cell damage.

MDA is one of the largest toxic effects produced by lipid peroxidation. Its level affects the rate and intensity of lipid peroxidation, indirectly responding to the extent of free radical damage. The products of lipid peroxidation migrate to unused sites in neurons, leading to a variety of deleterious changes that impair cellular function and ultimately lead to neuronal death. The process of lipid peroxidation is accompanied by the release of lactate dehydrogenase (LDH), a key anaerobic enzyme prevalent in cells, whose release directly reflects the violation of cell membrane integrity and the occurrence of abnormal oxidative effects and physiological metabolism. When the organism is exposed to oxidative stress damage, various endogenous antioxidant enzymes and antioxidant substances are produced to fight oxidative stress damage. SOD, CAT, and GSH-Px are specific sets of enzymes that work in coordination to prevent high intracellular levels of reactive oxygen species, also known as the protective enzyme system, and play an important role in the protection of neural cells from free radical damage (López et al., 2013). In this study, we showed that GPP pretreatment significantly inhibited H_2_O_2_-induced oxidative stress death in PC12 cells, attenuated the levels of lipid peroxidation MDA and ROS, slowed LDH leakage and increased the expression levels of endogenous antioxidant enzymes SOD, GSH-Px, and CAT.

It is well known that mitochondria play a key role in cell metabolism, including energy metabolism, fatty acid oxidation, calcium homeostasis and apoptosis. Neuron cells especially depend on the function of mitochondria, especially their energy supply, so the damage to mitochondrial function may seriously interfere with the physiological metabolism of neurons. Mitochondrial dysfunction in AD is associated with increased free radical production. The occurrence of mitochondrial dysfunction will lead to oxidative stress, reduced cytochrome c oxidase activity and decreased energy metabolism. MMP is an electrochemical potential formed between the two sides of the inner mitochondrial membrane during the process of respiratory oxidation, and a decrease in MMP is closely related to apoptosis (Anandatheerthavarada and Devi, 2007; Singulani et al., 2021). In this study, we used JC-1 staining to investigate the effect of H_2_O_2_ damage on the membrane potential of PC12 cells, and the results showed that H_2_O_2_-induced oxidative stress significantly decreased MMP, while GPP pretreatment enhanced cellular MMP to resist oxidative stress damage.

Neuroinflammation has been shown to be one of the factors contributing to the progression of AD, when the initial inflammatory stimulus (oxidative stress, Aβ, pathogenic infection, etc.) triggers the activation of central nervous system microglia (CNS). They secrete various pro-inflammatory cytokines (including IL-1β, IL-6, TNF-α and chemokines) will further recruit immune cells to the site of inflammation. Under normal conditions, this process is well controlled, and immune cells are recruited to the site of inflammation and facilitate the removal of the initial stimulus/pathogen, followed by the resolution of the inflammatory response (Minter et al., 2016). However, in AD, abnormally increased Aβ, abnormal Tau proteins and higher ROS levels force immune cells to fail to effectively clear inflammation, which leads to excessive production of pro-inflammatory and chemokines, inducing further expansion of neuroinflammation (Zhang and Jiang, 2015). In the present study, it was found that H_2_O_2_ injury increased the levels of cellular secretion of NO, TNF-α, and IL-6, indicating that the injury caused the recruitment of inflammatory factors, whereas GPP pretreatment significantly reduced the secretion of inflammatory factors in H_2_O_2_ injury and slowed the recruitment effect of neuroinflammation.

Accumulating evidence demonstrates that changes in brain metabolism associated with oxidative stress, neuroinflammation, and mitochondrial dysfunction play an important role in the pathophysiology of AD. For example, hypometabolism in AD-affected brain regions with altered mitochondrial structure and reduced expression and activity of mitochondrial enzymes critical for energy metabolism; decreased mitochondrial membrane potential and increased permeability of neuronal cells in AD lesion areas would induce excessive ROS production (Song et al., 2021). Furthermore, neuroinflammation mediated by microglia is now considered a hallmark feature of various neurodegenerative diseases (Calabrese et al., 2014). Notably, the expression of pro-inflammatory mediators is regulated by microglia mitochondrial dynamics, and neuroinflammation is also closely associated with OS, and increased neuroinflammation will further induce OS production (Verri et al., 2012). In conjunction with the research conducted, we found that GPP reversed H_2_O_2_-induced ROS production, IL-6, TNF-α, and NO inflammatory factor levels in PC12 cells, improving mitochondrial membrane potential, suggesting that GPP may act through multiple targets, systems and links, We also found that 200 μg/ml GPP pretreatment achieved a better protective effect, which is close to the effective concentrations reported in other studies on the effects of plant polyphenols in the treatment of AD (1000 μg/ml and 100 mg/kg for Black Chinese Wolfberry Polyphenols and Tea Polyphenols, respectively, in AD models reported in the literature) (Fengjun et al., 2010; Xiao and Qinghan, 2019). The above study found that plant polyphenols act through improving ROS levels, mitochondrial apoptosis and Caspase signaling apoptotic pathways in injury models. In summary, GPP may act through multiple targets, systems, and links, which is consistent with the therapeutic characteristics of TCM in dementia (Chen et al., 2020).

## 5 Conclusion

GPP, a polyphenolic component, was extracted and purified from the Chinese herb *G. elata* using an ultrasonic/microwave extractor for the first time. The effect of GPP pretreatment on the injury model was investigated using an H_2_O_2_-induced PC12 cell injury model simulating oxidative stress injury. In this study, we found that 50–200 μg/ml GPP pretreatment significantly improved cell survival, reduced LDH leakage and increased the levels of endogenous antioxidant enzymes (SOD, CAT, GSH-Px) in the injury model; meanwhile, GPP improved ROS, inflammation and apoptosis levels in the injury model, suggesting that GPP may function through the above mechanisms. Antioxidants exhibited promising protective effects in the H_2_O_2_-mediated oxidative stress model of Alzheimer’s disease. Notably, *G. elata* is approved by the Chinese Food and Drug Administration (CFDA) as a substance that can be used in healthy food and as a drug. The present study complements the anti-AD effect of its polyphenolic components and provides a reference for the future development of new drugs or functional health food products from *G. elata*.

## Data Availability

The original contributions presented in the study are included in the article/supplementary material, further inquiries can be directed to the corresponding author.
